# Native Variants of the MRB1 Complex Exhibit Specialized Functions in Kinetoplastid RNA Editing

**DOI:** 10.1371/journal.pone.0123441

**Published:** 2015-04-30

**Authors:** Bhaskara R. Madina, Vikas Kumar, Blaine H. M. Mooers, Jorge Cruz-Reyes

**Affiliations:** 1 Department of Biochemistry and Biophysics, Texas A&M University, College Station, TX 77843, United States of America; 2 Department of Biochemistry & Molecular Biology, University of Oklahoma Health Sciences Center, Oklahoma City, OK 73104, United States of America; University of Toronto, CANADA

## Abstract

Adaptation and survival of *Trypanosoma brucei* requires editing of mitochondrial mRNA by uridylate (U) insertion and deletion. Hundreds of small guide RNAs (gRNAs) direct the mRNA editing at over 3,000 sites. RNA editing is controlled during the life cycle but the regulation of substrate and stage specificity remains unknown. Editing progresses in the 3’ to 5’ direction along the pre-mRNA in blocks, each targeted by a unique gRNA. A critical editing factor is the mitochondrial RNA binding complex 1 (MRB1) that binds gRNA and transiently interacts with the catalytic RNA editing core complex (RECC). MRB1 is a large and dynamic complex that appears to be comprised of distinct but related subcomplexes (termed here MRBs). MRBs seem to share a ‘core’ complex of proteins but differ in the composition of the ‘variable’ proteins. Since some proteins associate transiently the MRBs remain imprecisely defined. MRB1 controls editing by unknown mechanisms, and the functional relevance of the different MRBs is unclear. We previously identified two distinct MRBs, and showed that they carry mRNAs that undergo editing. We proposed that editing takes place in the MRBs because MRBs stably associate with mRNA and gRNA but only transiently interact with RECC, which is RNA free. Here, we identify the first specialized functions in MRBs: 1) 3010-MRB is a major scaffold for RNA editing, and 2) REH2-MRB contains a critical trans-acting RNA helicase (REH2) that affects multiple steps of editing function in 3010-MRB. These trans effects of the REH2 include loading of unedited mRNA and editing in the first block and in subsequent blocks as editing progresses. REH2 binds its own MRB via RNA, and conserved domains in REH2 were critical for REH2 to associate with the RNA and protein components of its MRB. Importantly, REH2 associates with a ~30 kDa RNA-binding protein in a novel ~15S subcomplex in RNA-depleted mitochondria. We use these new results to update our model of MRB function and organization.

## Introduction

*Trypanosoma brucei* and other kinetoplastid protozoa have a single mitochondrion with an unusual mitochondrial genome (kDNA) consisting of many copies of an identical “maxicircle DNA” (~23 kb) and several hundred different types of “minicircle DNAs” (~1 kb). Maxicircle DNA encode 18 mRNAs, most of which require maturation through a remarkable form of RNA editing that changes the length of the mRNA by insertion or deletion of uridylate (U) nucleotides. In contrast, each minicircle DNA encodes 3–4 genes for small guide RNAs (gRNAs) that bind the pre-mRNA in *trans* and direct editing at more than 3000 sites [1,2]. gRNAs (~45–60 nt) are primary transcripts with a 3’ tail of ~10–15 Us added post-transcriptionally. Each gRNA has base-sequence complementary to the fully edited mRNA sequence [3]. RNA editing progresses from 3’ to 5’ along the mRNA through sets of overlapping sequence blocks; the editing of each block is directed by a different gRNA. This process also exhibits substrate specificity during the life cycle, but the mechanisms involved remain unknown [4]. Most mRNAs are extensively edited, while a few require limited editing or are never edited [5]. This process often creates start or stop codons, and often generates most of the open reading frame. A mistaken insertion or deletion of a single U creates a frameshift in the mRNA and could create a nonfunctional protein during translation. Surprisingly, mitochondria contain many partially edited mRNA transcripts. Partial editing is always located in a junction in the RNA between the 5’ unedited and 3’ fully edited sections [6,7].

The editing enzyme, known as the RNA editing core complexes “RECC” or editosomes, has been extensively studied [4,8,9], but many central questions remain unanswered. For example, how are the substrates recruited, and how are editing initiation and progression controlled? This is particularly puzzling because the purified RECC enzyme lacks the editing processivity found *in vivo* [10–12] and early studies showed that it does not contain endogenous gRNA or mRNA [13]. A substantial number of non-RECC proteins that control editing have been reported [4,14,15]. Several of these latter proteins are components of a large accessory complex, MRB1, which binds and stabilizes gRNA, and transiently interacts with the RECC [16,17]. Most proposed MRB1 subunits lack recognizable sequence motifs, reflecting the early phylogenetic divergence of kinetoplastids [18]. Some proteins may be part of a putative core in MRB1, while others appear to be variable components. The functional relevance of the variation in the composition of the MRBs remains unclear [19,20]. Inducible RNAi of select MRB1 proteins have suggested effects in editing at an early stage or during progression [19–21]. Certain proteins primarily affect specific mRNAs, while others have broader effects [17,22], implying substrate preferences. We have reported two native MRB1 variants (termed here “MRBs”), REH2-MRB and 3010-MRB, with clear differences in protein and RNA composition. Importantly, these MRBs carry unedited and fully edited mRNAs, in addition to gRNA. Our findings suggested that these complexes assemble mRNA-gRNA hybrids and raised the possibility that different MRBs exhibit specialized functions [23]. A more recent report from another lab confirmed that MRB1 contains mRNAs [24]. REH2-MRB and 3010-MRB have a differential partition of several initiating gRNAs and of the RNA helicase REH2 and MRB3010. RNAi knockdown of either protein inhibits editing and the latter was proposed to be important in early editing [19,20]. Notably, deep sequencing studies in total mtRNA in the Koslowsky’s lab and in purified MRBs in our lab found that most initiating gRNAs are rare [23,25]. These results suggest that the initiation of editing may be regulated.

In our model of MRB1 function and organization [23], that we further test here, MRB1 variants serve as scaffolds for the assembly of mRNA-gRNA hybrids and the RECC enzyme, and these variants can be linked to specific roles in RNA editing. Specifically, we hypothesized that 3010-MRB supports efficient editing initiation and that REH2 is a trans-acting factor of 3010-MRB. Our findings indicate that 3010-MRB is relatively enriched with mRNAs that are edited at the first block directed by the initiating gRNA and that REH2 affects multiple editing steps in mRNAs associated with 3010-MRB. Furthermore, REH2 binds its native MRB via RNA, and point mutations in conserved motifs of the helicase inhibit its association with RNA and protein components of the REH2-MRB. Finding functionally distinct MRBs that include regulatory proteins and all mRNAs involved in editing, i.e., unedited, partially edited and fully edited transcripts, raises a number of important mechanistic questions that can now be directly addressed in the RNA editing of early-branched kinetoplastids.

## Materials and Methods

### Cell culture

*T*. *brucei* Lister strain 427 29–13 procyclic “PF” (tryps.rockefeller.edu) was grown axenically in log phase in SDM79 medium [26] and harvested at a cell density of 1-3x10^7^ cells/ml. Cell lines expressing TAP-REH2 variants or a construct for REH2 down-regulation were induced with tetracycline at 1 μg/ml.

### REH2 plasmid constructs

We introduced point mutations K1078A and A1086D in the double-stranded RNA binding domain (dsRBD) of REH2 by PCR-based site-directed mutagenesis of a pLew79 TAP-REH2 construct [19] using a proofreading thermostable polymerase mix (AccuTaq, Sigma) and oligonucleotides described in S1 Table. We made an RNAi construct by PCR amplification of a 344-bp fragment from the REH2 3’UTR region using oligonucleotides described in S1 Table, and we cloned this fragment into the XhoI and BamHI sites of p2T7-177 [27]. All constructs were confirmed by DNA sequencing, linearized with NotI, and transfected in procyclic 29–13 trypanosomes [26].

### Protein and RNA purification from the pulldowns

We performed immunoprecipitation of REH2, MRB3010, and cytochrome oxidase 2 (mock) using affinity-purified peptide antibodies as described [23]. Briefly, specific antibodies were conjugated to Dynabeads Protein A (Life Technologies) that were pre-treated with 5% BSA. Approximately 2 mg of mitochondrial extract was supplemented with 1X Complete Protease Inhibitor cocktail (Roche) and SUPERase·In RNase inhibitor (Life Technologies). The extract was pre-cleared by passage over Protein A-Sepharose beads (GE Healthcare) before it was loaded onto antibody-conjugated beads. Ectopically expressed TAP-REH2 was specifically immunopurified using Dynabeads IgG (Life Technologies), as reported elsewhere [19]. All washes were performed with 200 mM NaCl, 1 mM EDTA, 10 mM MgCl_2_, and 25 mM Tris, pH 8. Protein was extracted with 1X SDS loading buffer at 95°C for 2 min. RNA was extracted by treating the beads with 0.8U proteinase K (NEB) for 30 min at 55°C, followed by phenol extraction and ethanol precipitation.

### Glycerol gradients, western and northern blots, and radioactive assays

Sedimentation fractions were obtained from freshly made mitochondrial extracts in 10–30% glycerol gradients [19]. Western blots of REH2, MRB3010 (3010), GAP1, and MP63 (a RECC subunit used as a ~20S marker) were performed as reported [19,28]. Crosslinking assays used gRNA gA6 B1.alt [23] as a model initiating gRNA bearing a photo-reactive 4thio-U and ^32^P that was mixed with immunopurified MRBs and subjected to 365 nm UV irradiation on ice as previously described [29,30]. The photo-reactive gRNA was prepared by a splint ligation with the oligonucleotides described in Table SI [30]. Radioactive capping of gRNA used RNA extracted from the Dynabeads protein A pulldowns [19]. Northern blots of total mitochondrial RNA or IPs from mitochondrial lysate, extracted using TRizol LS reagent (Life Technologies) and proteinase K (NEB), respectively, used 5’ labeled probes for the initiating gRNAs gND7 3’ domain B1, gCyB B1 [23,31], and for tRNA-Cys (S1 Table). These procedures were performed in ULTRAhyb solution (Life Technologies) with 2X SSC washes at 40°C.

### Quantitative RT-qPCR and endpoint RT-PCR of mRNAs

RNA from pulldowns was treated with RNase-free DNase (Thermo) and used in the preparation of cDNA as described elsewhere [23]. RT-qPCR assays normalized using the delta delta CT “ddCT” (Livak) method [8,32] were performed in 20 microliter reactions with the primers reported to be specific for unedited mRNAs, edited at a 5’ distal block, and reference transcripts [8], or primers designed by us in the present study to analyze early 3’ editing (S1 Table) in a SYBR Green PCR Master Mix (Bio-Rad). RT-PCR of RPS12, A6, and ND7 fragments was performed using PerfeCTa SYBR Green FastMix (Quanta) with specific oligonucleotides with unedited or never-edited sequences (S1 Table), and analyzed on 8% native acrylamide gels. All amplicons in this study were verified by cloning and manual sequencing. cDNA at different dilutions and no-RT controls were tested to confirm the linearity and specificity of the amplifications.

### Cloning and sequencing of mRNA block 1 sequence

RNA extracted from REH2 and 3010 antibody pulldowns was C-tailed with Poly A Polymerase (NEB) and CTP. The C-tailed RNAs were used for cDNA synthesis with Superscript III reverse transcriptase (Invitrogen) and a 3’ polyG-ended RT primer (S1 Table). This cDNA was amplified with the RT primer and a transcript-specific oligonucleotide using Taq polymerase (NEB). PCR products were isolated from a 10% native PAGE and re-amplified by nested PCR (S1 Table). The final amplicons were analyzed on 10% native acrylamide gel or were gel isolated, cloned (StrataClone PCR Cloning Kit, Agilent), and sequenced.

### Structural analyses of REH2

Domain annotations in REH2 including the previously unidentified OB-fold domain were performed using the Conserved Domain Search tool (CD-Search) at NCBI [33]. The mot I mutations were modeled using a homology model of the helicase portion (residues 1308 to 1846) of *T*. *brucei* REH2. The coordinates of the ADP were from the crystal structure of a yeast Prp43p/ADP complex (2XAU) [27] after superposition of the crystal structure of the complex onto the homology model of REH2 which had been made with Phyre2 [34]. The mutations were made using the backbone dependent rotamer library [35] in PyMOL. The selected glutamine rotamer was the only rotamer that lacked steric clashes and that had favorable interactions with the surrounding atoms. The distances are in ångstroms.

## Results

### 3010-MRB associates with mRNAs that exhibit efficient editing at block 1 directed by the initiating gRNA

**Fig 1:**
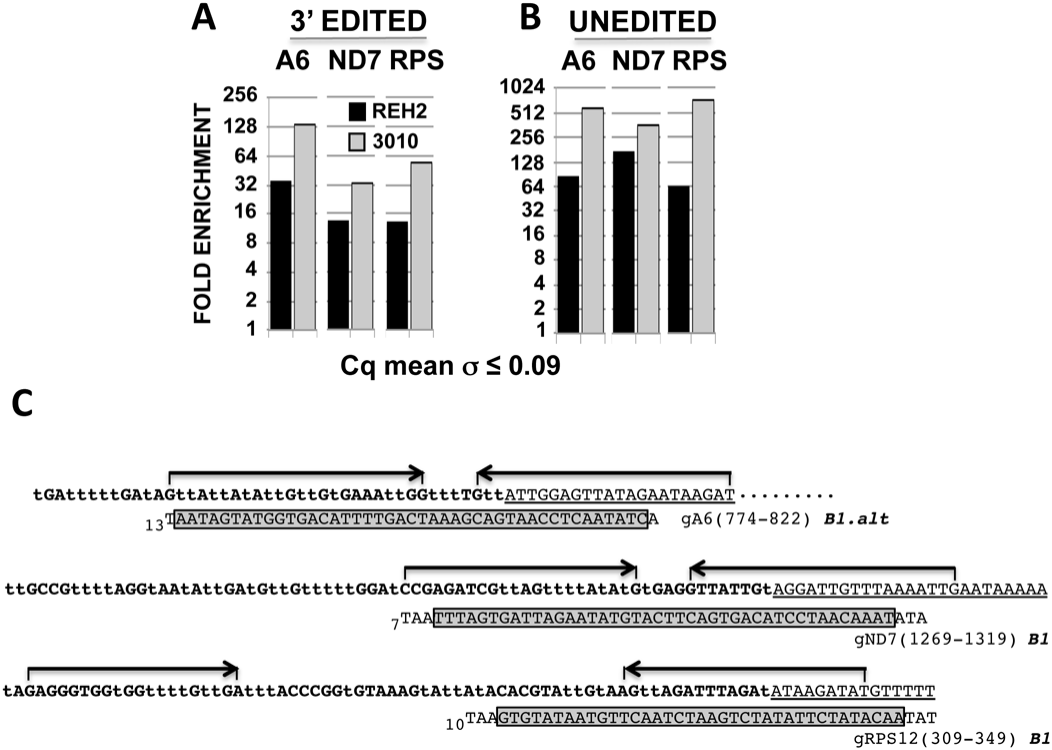
Quantitative analysis of early 3’ edited and unedited mRNAs. (A) Enrichment of edited block 1 in mRNAs A6 and ND7, or the first few blocks in RPS12, in 3010 and REH2 IPs relative to a mock IP set at 1. RT-qPCR values of test IPs were normalized to input values and to a low nuclear 18S rRNA carryover in the beads as loading control. [Fold = 2^*ddCq*^, in which *ddCq = IP dCq – Input dCq*, and *dCq = test Cq – 18S Cq*. Cq is the quantification cycle.]. Standard deviation of the average value of Cq duplicates is shown. All end-point amplicons were single products during linear amplification, and sequenced. (B) Relative fold enrichment of unedited pre-mRNA calculated as in panel B. (C) Amplified edited sequence in panel A. Edited 3’ sequence in mRNAs A6, ND7, and RPS12 is aligned with a major initiating gRNA (in 3’→5’ orientation) [23,25]. Regions of interest are identified as follows: mRNA editing domain (bold), never-edited sequence (underlined), gRNA guide domain (gray box), and length of the 3’ U tail (subscript). PCR primers (arrows) were designed based on the sequenced 3’ end of cloned cDNA fragments (Fig 2 and S1 Fig, and data not shown). The U-insertions (“t”) and deletions (not shown) allow high-quality duplexes with the guide domain of initiating gRNAs. All amplicons were cloned and sequenced. A previously annotated encoded T at position 1269 in block 1 of mRNA ND7 is not included because it was missing in the sequenced unedited transcripts from the Lister strain in this study (S1 Fig).

**Fig 2:**
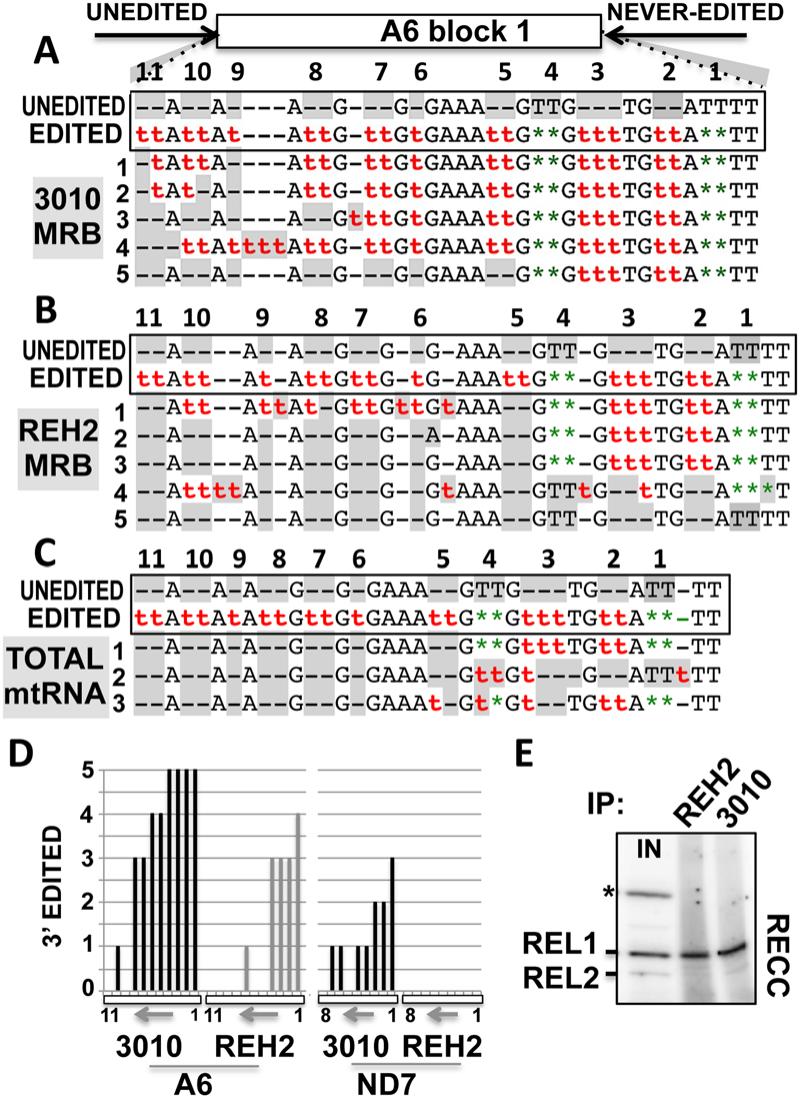
Efficient editing at block 1 in mRNAs bound to 3010-MRB. Block 1 sequence of a few mA6 transcripts amplified from (A) 3010 and (B) REH2 IPs or (C) total mtRNA from the mitochondrial extract loaded in the IPs. PCR primers (arrows) target 5’ unedited and 3’ never-edited sequence. The sequences in a box show sites 1-to-11 either unedited (gray) or fully edited with insertions ‘t‘ and deletions ‘★’. In clones 1-to-5, misediting (in number or site) is also shown in gray. (D) Number of correctly edited sites in the randomly selected clones of mRNAs A6 and ND7 (11 and 8 sites, respectively) purified from the IPs in panels A and B above and S1 Fig. Each bar is one site. The 3’-5’ direction of editing (arrow) in block 1 (box) is indicated. (E) Radioactive autoadenylation of ligases REL1 and 2 in the RECC enzyme detected in REH2 and 3010 IPs. An asterisk marks a contaminant cytosolic ligase in mitochondrial extract input (IN). Pre-charged REL2 with endogenous ATP is poorly radiolabeled [45].

We tested whether or not 3010-MRB and REH2-MRB are functionally different because they have distinct protein compositions and carry unedited and fully edited RNAs, and because 3010-MRB is enriched in initiating gRNAs [23]. We began by comparing the efficiency of editing directed by the initiating gRNA in mRNAs associated with 3010 and REH2 MRBs. To this end, we established quantitative RT-PCR (RT-qPCR) assays for block 1 in mRNAs A6 (ATPase subunit 6) and ND7 (NADH dehydrogenase subunit 7) or the first few blocks in mRNA RPS12 (ribosomal protein subunit 12) (Fig 1). Our assays were based on confirmed 3’ edited sequence described below (Fig 2 and S1 Fig, and additional data not shown) and the recently identified gRNAs [23,25].

The results from these assays showed a greater concentration of block 1 edited mRNA in 3010-MRB than in REH2-MRB (Fig 1A). We also scored unedited transcripts and found that the three tested mRNAs were enriched in 3010-MRB (Fig 1B), consistent with our previous study [23]. Interestingly, the A6 edited pattern in block 1, which is located in the mRNA 3’ UTR, matched nearly the entire initiating gRNA that was recently identified in two strains of procyclic trypanosomes (Fig 1C) [23,25]. Both edited block 1 and the initiating gRNA differed substantially from those originally reported in the 1990s [3], indicating a rapid evolution of the responsible minicircle(s). The sequence of the 3’ edited mRNA ND7 was as reported earlier, except for one residue (S1 Fig). The first few blocks in mRNA RPS12 that we examined were also as originally reported. The edited sequence for block 2 in mRNA RPS12 was surprising because the guiding potential of a major gRNA in the Lister strain predicted an alternative editing pattern [23]. Thus, the tested block 1 edited mRNAs and their unedited substrates are relatively more abundant in 3010-MRB than in REH2-MRB.

To determine if 3010 and REH2 MRB complexes carry mRNA intermediates with partial editing directed by the initiating gRNA, we sequenced the editing block 1 in A6 and ND7 mRNAs isolated from the complexes or from total mitochondrial mRNA (mtRNA) (Fig 2A–2C, and S1 Fig). Briefly, we amplified the cDNAs with primers containing unedited sequences just 5’ of block 1 and never-edited sequences just 3’ of the first editing site. This sequencing strategy allowed us to survey the first few editing sites for editing efficiency. Although we analyzed only a few clones, it was clear that both 3010 and REH2 MRBs carry RNAs with partial editing at block 1. Interestingly, the examined transcripts from 3010-MRB exhibited more complete editing in the first sites than transcripts from REH2-MRB and the mtRNA population. A count of the edited sites in the clones analyzed above is shown in Fig 2D. Interestingly, editing in ND7 was not as extensive as it was in mRNA A6, suggesting a substrate preference. In fact, the sequenced ND7 transcripts from the REH2-MRB complex were unedited, consistent with the reported ratio of unedited to edited mRNA ND7 in this complex [23]. Importantly, purified 3010 and REH2 MRBs in our experiments associate with comparable amounts of RECC (Fig 2E). Thus, both MRB complexes associate with the editing enzyme and contain all mRNAs involved in editing, i.e., substrates, intermediates and products, indicating that both 3010 and REH2 MRB complexes are competent editing scaffolds. However, the above data together with our previous report [23] also indicate that the 3010-MRB complex is enriched in mRNAs with efficient editing at block 1 and the corresponding initiating gRNAs and pre-mRNA substrates.

### REH2 affects the level of unedited pre-mRNA, and editing at block 1 and at upstream blocks in 3010-MRB

**Fig 3:**
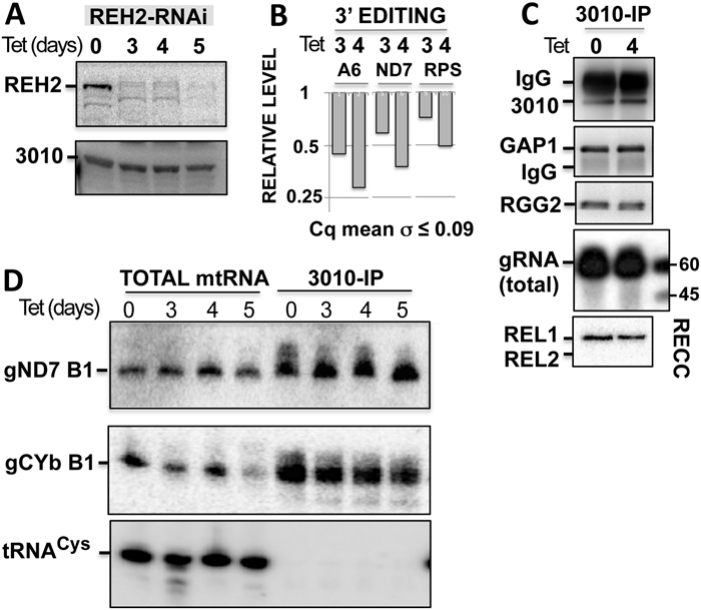
REH2 knockdown affects editing by the initiating gRNA in 3010-MRB but not 3010 association with common MRB1 proteins, gRNA or RECC. (A) Western blots of REH2 and 3010 in lysates of induced cells with tetracycline (Tet). Full length and proteolytic fragments of REH2 are detected in all Figs in this study. (B) Relative level of early 3’ editing in block 1 of mRNAs A6, ND7, and the first few blocks in RPS12 (RPS). RT-qPCR assays were performed in cell lysates using tubulin as the reference. Uninduced samples are set at 1. (C) 3010-IPs of mitochondrial extracts at 0 or 4 days post-induction in western blots of 3010 and GAP1, or radiolabeled capping (gRNA) and autoadenylation (editing ligases REL1/2). (D) Northern blots of initiating gRNAs for block 1 (B1), gND7 (1269–1319), and gCYb gCYb (54–91) in total mtRNA and 3010-IPs at multiple time points of RNAi induction. The blots were stripped and reprobed for tRNA-Cys, showing that the pulldowns are specific for gRNA.

Although REH2 and 3010 are subunits of different MRBs, we asked if the REH2 helicase affects *in trans* the function or composition of the 3010-MRB complex. For example, REH2 may impact the initiation or progression stage of editing in 3010-MRB, or REH2 may impact the association of the substrate pre-mRNAs with this complex. Inducible RNAi of REH2 inhibited editing at early 3’ sites on pre-mRNA substrates in total mtRNA (Fig 3A and 3B), but this REH2 depletion did not affect the cellular level of 3010 or the copurification of 3010 with the core GAP1 subunit, with RGG2 (another common protein in MRB purifications), total gRNA, or the RECC enzyme (Fig 3C). Northern blot analyses showed that the level of initiating gRNA gND7 in the total mtRNA and in the 3010-MRB complex was not substantially affected by depletion of REH2 (Fig 3D). A somewhat decreased level of the initiating gRNA (gCYb B1) in the complex is puzzling. A recent study suggested that gRNA recycling during editing leads to accumulation of gRNAs upon editing inhibition [24]. In summary, these results indicate that the editing directed by the initiating gRNA requires REH2.

**Fig 4:**
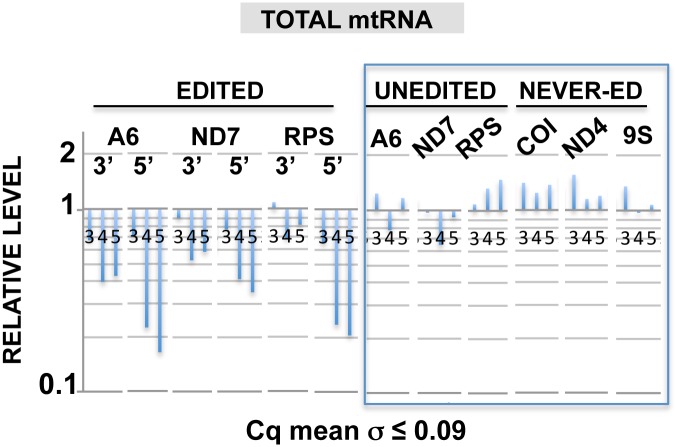
REH2 knockdown affects editing progression. (A) RT-qPCR of steady-state edited mRNA at block 1 in mRNAs A6 and ND7, and the first few blocks in RPS12 (3’ sites) or a distal block (5’ sites), unedited or never-edted RNAs at days 3, 4, and 5 of REH2 RNAi. Uninduced samples are set at 1.

**Fig 5:**
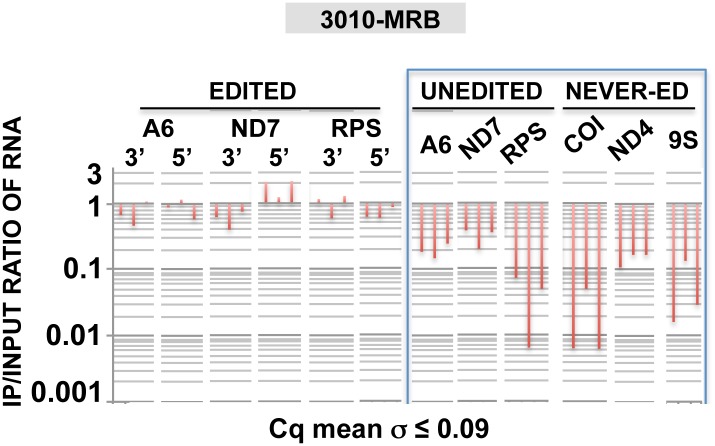
REH2 knockdown decreases the content of unedited pre-mRNAs in 3010-MRB. The ratio of select transcripts in IP samples and mitochondrial extract input (IP/input) was determined. Each RNA transcript was scored by RT-qPCR at days 3, 4 and 5 of REH2 RNAi. Uninduced ratios are set at 1. Standard deviation of the average value of Cq duplicates is shown. All amplicons were validated by cloning and sequencing. 18S rRNA in lysates and beads was used as reference.

Further analyses in mtRNA compared editing at early 3’ sites and at a distal block sequence (late 5’ sites) and showed greater inhibition of editing at the late sites (Fig 4). This suggests that editing progression across multiple blocks also requires REH2. In contrast, the levels of the unedited and never-edited mRNAs, and mitochondrial rRNA 9S remained relatively constant. Thus, REH2 depletion did not significantly affect unedited pre-mRNA at steady state. REH2 could, however, affect the level of unedited pre-mRNA in the 3010-MRB complex. To assess RNA association with the complex, we determined the ratio of mRNA in the 3010-IP and mitochondrial lysate input, i.e., IP/input (Fig 5). That is, regardless of the steady state level of pre-mRNA substrate (rather stable) and edited transcripts (decreased) upon REH2 depletion, we asked whether or not the proportion of molecules in the 3010-MRB complex and in the total mtRNA population is maintained. Notably, REH2 ablation decreased the ratio of unedited substrates in the 3010-MRB complex between 7 and over 10 fold at different time points of induction, particularly with RPS12. This change in the level of unedited pre-mRNA in the complex was confirmed by gel analysis of semi-quantitative RT-PCR amplicons (data not shown). In contrast, the ratio of edited RNA in the complex that we scored at early 3’ sites or at a distal 5’ block was maintained during the REH2 knockdown (Fig 5). Thus, while REH2 depletion decreased significantly the total amount of edited mRNA in the parasite, it did not significantly affect the ratio of associated edited molecules with the complex. This effect was consistently observed at multiple time points of the REH2 RNAi induction. Moreover, all observations can be made at the shortest time point (day 3) when the growth phenotype is first detected (not shown). We controlled for secondary effects by showing normal steady state levels of MRB1 markers (e.g., 3010 and gRNA) and several mitochondrial transcripts in the total mtRNA pool (unedited and never edited mRNA, gRNAs, tRNA and 9S rRNA) at all time points of induction in these studies (Figs 3 and 4).

Interestingly, REH2 depletion also caused a substantial loss of never-edited mRNA and 9S rRNA in the 3010 pulldowns indicating a decreased association of the purified 3010-MRB with mitoribosomes [23]. Never-edited mRNA is thought to directly bind ribosomes [36]. Thus, depletion of REH2 helicase appears to affect the substrate content of the 3010-MRB as well as the association of this complex with mitoribosomes.

**Fig 6:**
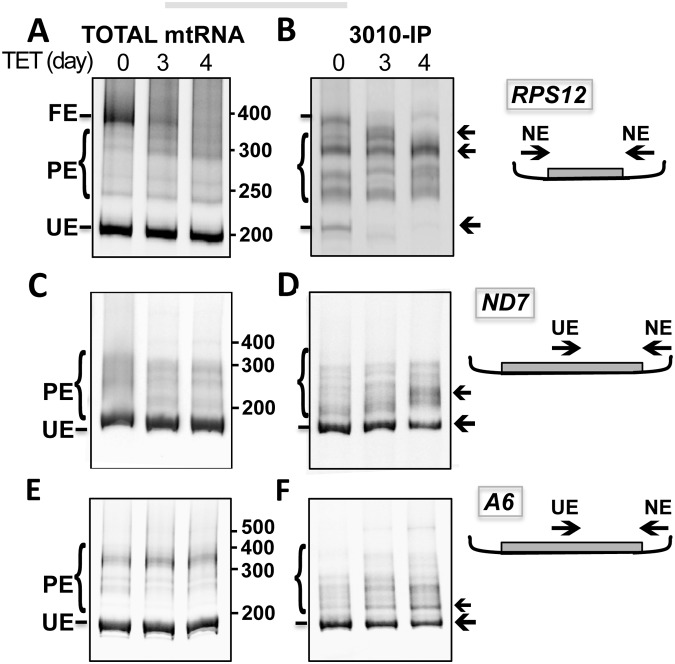
REH2 knockdown decreased substrate loading and increased pausing during editing in 3010-MRB. Endpoint RT-PCR products in total mtRNA and 3010-IPs at 0, 3, and 4 days of REH2 RNAi. mRNAs RPS12 (A and B), ND7 (C and D) or A6 (E and F). The full editing domain in RPS12 or a 3’ fragment of similar size in ND7 and A6 was amplified. Products of varying sizes are unedited (UE), partially edited (PE), or fully edited (FE), in the case of RPS12. Arrows point to regions of apparent major pausing induced by REH2 depletion. The primers target 5’ unedited (UE) or 3’ never-edited (NE) sequences, as depicted in the cartoons.

The pool of transcripts in the mitochondrion includes unedited, fully edited, and partially edited RNAs of different sizes. To further analyze the REH2 effects, we amplified the entire ~200 nt mRNA RPS12 using primers targeting never-edited sequences at the termini of the transcript, and we visualized the products in a gel (Fig 6A and 6B). In total mtRNA from REH2 knockdown cells, the amount of fully edited RNA decreased, while the amount of unedited RNA remained fairly constant (Fig 6A). However, in the purified 3010-MRB, the amount of unedited RNA decreased (Fig 6B). Furthermore, the complex accumulated partially edited molecules of various sizes in a pattern suggesting preferential sites for pausing. These findings support the idea that REH2 acts at multiple editing steps in pre-mRNAs associated with 3010-MRB. Analysis of mRNAs ND7 and A6 yielded similar results (Fig 6C, 6D, and 6E, 6F, respectively, and additional data not shown). However, these effects were particularly clear with RPS12 consistent with our data in Fig 5. Also, because the ND7 and A6 substrates are much longer, we only examined a 3’ fragment (~200 nt), including the first editing blocks, to analyze unedited and partially edited sequences. In summary, our data indicate that REH2 has multiple editing roles including in substrate loading, editing by the initiating gRNA and editing progression in upstream blocks.

### REH2 binds its native MRB via RNA, and can be purified in a novel ~15S RNA-free subcomplex

Although native REH2 and 3010 are found in large ribonucleoprotein MRBs, the nature of the stable association of REH2 with other MRB1 proteins is unclear. A previous purification of REH2 contained core GAP subunits (GAP1/2), 3010, and other known MRB1 proteins [19]. Conversely, purifications of GAP subunits contained REH2 [16,19]. Some of these purifications included an extensive RNase treatment in attempts to remove RNA-mediated associations in the complexes. Interestingly, a yeast two-hybrid screen of several MRB1 proteins did not detect interactions with a REH2 fusion [37]. We further examined the REH2 interaction with GAP1 in the native REH2-MRB. Because RNA-mediated associations in MRBs may partially resist RNase attack, we stopped RNA production in mitochondria by knocking down its single RNA polymerase (RNAP) in a procyclic cell line [38].

**Fig 7:**
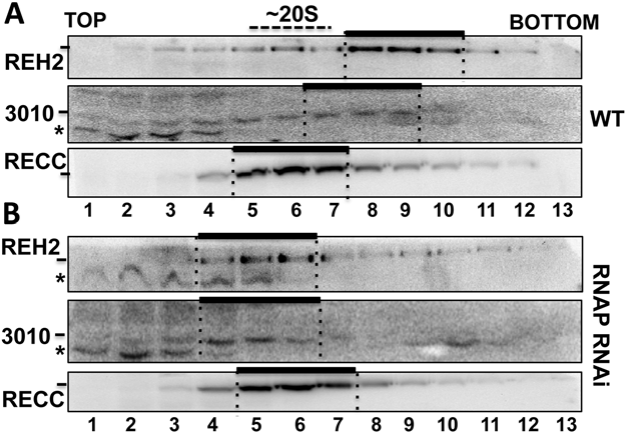
Sedimentation of MRBs. REH2 and 3010 MRBs in 10–30% glycerol gradients of mitochondrial extract from (A) wild-type (WT) or (B) RNAP knockdown cells at day 3 post-induction. The RECC complex (MB63 subunit) is at ~20S in panels A and B. Some proteolysis (*) occurs in the top fractions. Bars mark major peaks of REH2, 3010, and RECC examined in western blots.

**Fig 8:**
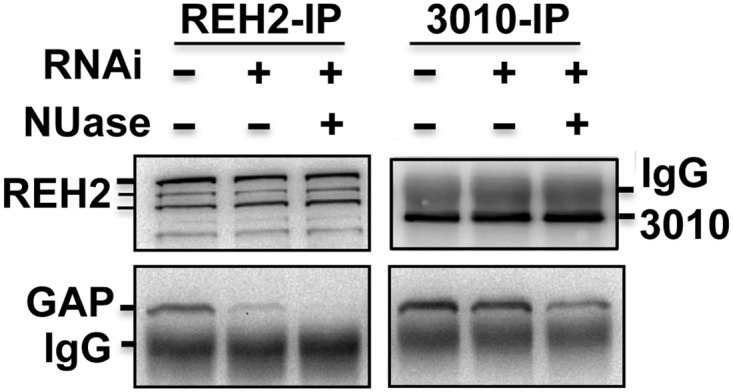
REH2 associates with GAP1 via RNA. Western blots of REH2, 3010, and GAP1 in IPs from RNAP knockdown cells, with or without a cocktail of nucleases (NUase): RNases A, and T1 and micrococcal nuclease. Untreated mitochondrial extract was used as a control.

Native MRBs are heterodispersed in sedimentation gradients (Fig 7A). However, depletion of most mitochondrial RNA (not shown) [38] significantly reduced the sedimentation peak of REH2 and 3010 to fractions slightly lighter than RECC at ~20S [13], which we estimate to be near 15S (Fig 7B). The unaffected migration of RECC, which seems largely RNA-free in its purified form [13], served as a cell quality control. REH2 and 3010 IPs from mitochondrial lysates of the RNAP knockdown cells were examined for the presence of the GAP1 core protein. Notably, REH2 copurification with GAP1 was nearly lost (Fig 8), and RNase-treatment of the IP sample rendered the interaction undetectable. In contrast, 3010 copurified with GAP1 in all tested conditions. Thus, REH2 binds GAP1 via RNA.

**Fig 9:**
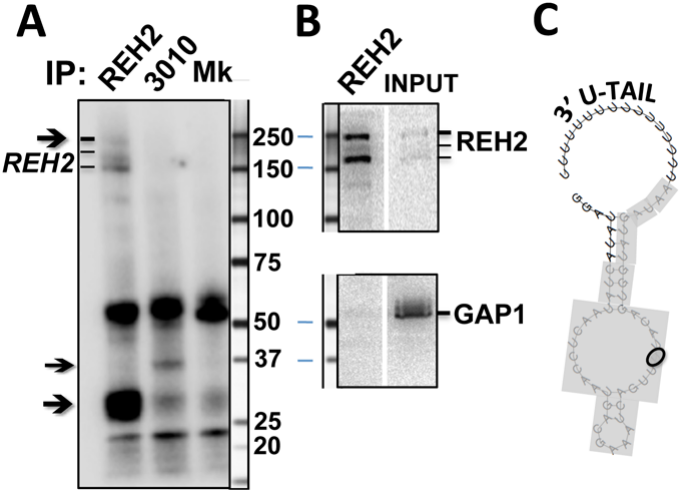
Photo-crosslinks in REH2 and 3010 pulldowns with an initiating gRNA. Antibody pulldowns of sedimentation fractions 4-to-6 in mitochondrial extracts from RNAP knockdown cells (Fig 7B) analyzed by (A) Site-specific crosslinking (365-nm UV) with a model initiating gRNA for mRNA A6 that includes a photo-reactive thio-U and ^32^P in a single phosphodiester bond, or (B) Western blots of REH2 or GAP1 with size markers in the REH2 IP or the 15S input fractions 4-to-6. (C) Mfold prediction [46] of the secondary structure of the gRNA in panel A. The photo-reactive base is circled and guide sequence is inclosed in the gray box. Arrows in A indicate crosslinks in the REH2 or 3010 IP, including at ~30 kDa and, apparently, REH2 itself in the REH2 IP. A mock control (Mk) used an irrelevant affinity-purified antibody.

We previously showed that REH2 and other presumed components of REH2-MRB in normal cells were able to photocrosslink with a 3’ fragment of A6 mRNA [19]. In the present study, we compared IP samples from the peak sedimentation fractions 4–6 (Fig 7B) from RNAP knockdown extracts in a photocrosslinking assay with a model initiating A6 gRNA (Fig 9A). Notably, we detected distinct crosslinks in the isolated REH2 and 3010 MRBs, which were absent in a mock purification. The REH2-associated crosslinks included a crosslink at ~30 kDa and weaker crosslinks at ~250 kDa, most likely involving REH2 itself because these crosslinks comigrate with REH2 in western blots (Fig 9B). Also, all other known protein subunits of MRB1 are much smaller than REH2. Recombinant GAP1 is known to bind a synthetic gRNA [16] but it is unclear if it photocrosslinks with our RNA probes. The crosslinks that we detected were stably bound because the samples in the beads had been treated with RNases and washed with 200 mM KCl. Together, our previous studies [23] and these new data indicate that native REH2 stably associates with its MRB via RNA, binds to both model mRNA and gRNA transcripts, and can be further purified together with a ~30 kDa RNA-binding cofactor in a novel ~15S “RNA-free” particle.

### Site-directed mutagenesis of conserved REH2 domains prevents the association of REH2 with GAP1 and editing substrates

**Fig 10:**
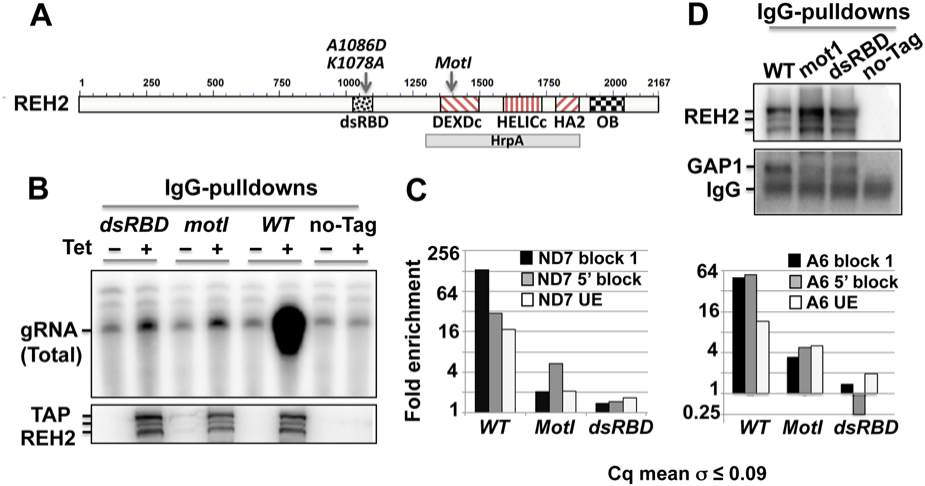
Association of REH2 with components of its MRB requires normal conserved domains. (A) REH2 (2,167 amino acids) includes the double-stranded RNA binding (dsRBD) and DExH-helicase domains, and an OB-fold domain not previously identified. Expressed TAP-tagged REH2 variants in Dynabead IgG pulldowns were tested for (B) gRNA in radiolabeled capping assays of the 5’ triphosphate, (C) fold enrichment of edited mRNA at block 1 or at a distal block, and unedited pre-mRNA. RT-qPCR assays were normalized to values in the untagged REH2 pulldown set at 1. Tubulin and 18S rRNA carryover were used as reference, (D) GAP1 and REH2 in western blots. Cells-/+ Tet induction of wild-type (WT), mutants, or untagged REH2 (no-Tag) were compared. Standard deviation of the average value in Cq duplicates is shown.

Because REH2 binds its native MRB via RNA, we examined the importance of the REH2 structure in these interactions. REH2 is a large (2,167 residues), non-ring-forming helicase (Fig 10A) that belongs to the RHA subfamily of the superfamily 2 (SF2) DEAH/RHA RNA helicases [39,40]. SF1 and SF2 helicases have a catalytic core of tandem RecA-like domains with characteristic motifs (I-VI) that participate in ATP-binding (S2 Fig) and hydrolysis and in RNA binding and unwinding. Accessory domains flanking the catalytic core determine their diverse functions by interacting with specific RNAs and proteins that modulate their activity [41,42]. RHA subfamily members, including REH2, have a unique conserved C-terminal region following the helicase core that contains an oligonucleotide-binding domain (OB-fold domain). A few subfamily members, including REH2, contain a ~70 residue double-stranded RNA-binding domain (dsRBD). The REH2 domain organization is conserved in kinetoplastids, including species of *Trypanosoma and Leishmania* (data not shown). Using expressed tagged constructs, we had shown that mutant REH2 proteins with either the dsRBD domain deleted or with two residue changes, G1365A/K1366Q, in the helicase motif I (mot I) were unable to copurify with gRNA [19]. We then tested the effect of mot I, and alanine substitutions of two highly conserved residues (K1078, D1086) [43] in the dsRBD on the normal interactions of REH2 (*cis* effects) in its MRB. The mot I and dsRBD point mutations inhibited REH2 copurification with editing substrates (gRNA and unedited mRNAs) and edited mRNAs at block 1 or at 5’ distal blocks (Fig 10B and 10C), and GAP1 (Fig 10D). Notably, homology modeling of the motif I or P loop using for the template the closest RHA subfamily member that has a published crystal structure with ADP bound [42] indicates that the mot I mutation removed a salt bridge (a H-bond plus ion-ion interaction) between the beta phosphate of the adenosine nucleotide and K1366 of REH2 (S2 Fig). This mutation would weaken the binding energy of ADP with the motif I through the loss of the counter ion and add the large energetic penalty of burying a negative charge without a counter ion. The beta phosphate would be left with four H-bonds. The alpha phosphate lacks a stabilizing salt bridge with a positively charged side-chain, so the protein will have difficulty compensating for the loss of the positive charge at site 1366 while continuing to counter the two negative charges of the ADP. The alpha phosphate may form two H-bonds (one with the backbone amide of T1386 and one with the side-chain hydroxyl of T1386), so the alpha phosphate contributes much less to the binding by ADP.

Overall, the above observations indicate that the integrity of native REH2-MRB, which includes GAP1 but not 3010, requires functional catalytic and RNA binding domains of REH2. These findings further suggest that the “RNA linkers” in REH2-MRB are in fact mRNA, gRNA, or mRNA-gRNA complexes.

## Discussion

MRB1 is a large and dynamic ribonucleoprotein complex that binds gRNA and is critical in the control of kinetoplastid RNA editing. However, MRB1’s specific molecular mechanisms of action and the rationale for MRB1’s dynamic composition are unclear. Our previous report [23] supported by the current study offer a novel conceptual framework proposing that editing is controlled and regulated in the context of at least two substrate-loaded MRB1 variants with specialized functions: 3010-MRB and REH2-MRB complexes. So far, the dissection of the function of specific editing proteins almost exclusively relied on RNAi knockdowns of the protein, followed by the analyses of several editing substrates. Also, early studies showed that RECC enzyme does not contain editing substrates [13]. So, long-standing questions in the field include: how does RECC access the editing substrates and how is this enzyme controlled? Our studies offer a path to systematically address the physical and functional interplay between the RECC editing enzyme, editing substrates and accessory MRB1 complexes.

**Fig 11:**
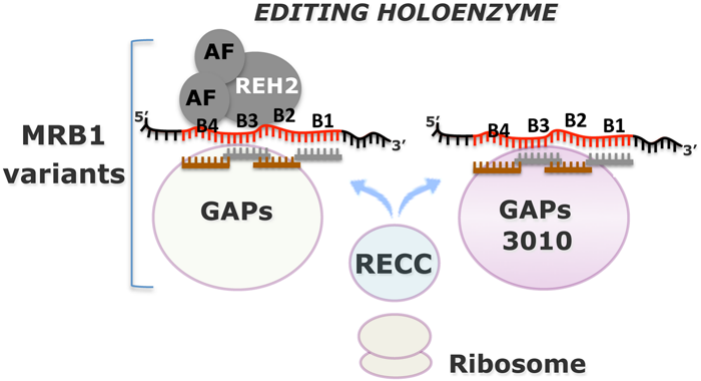
Model of MRB1 function and organization. MRB1 complexes carry editing substrates (pre-mRNA and gRNA), partially-edited intermediates and fully-edited mRNA, core GAP1/GAP2 proteins (GAPs), and other common and variable proteins. Addition of the RECC enzyme to MRB1 scaffolds forms the editing holoenzyme. Two MRB1 variants, REH2-MRB and 3010-MRB are structurally and functionally distinct. 3010-MRB appears to be a more active editing scaffold than REH2-MRB. REH2-MRB includes the regulatory REH2 helicase that affects 3010-MRB at multiple steps: mRNA substrate loading, and maturation at block 1 and subsequent blocks during editing progression. REH2 binds RNA and REH2’s association with other MRB1 components is mediated by bridging RNA and the helicase catalytic and RNA-binding domains. REH2 can be further isolated in a “RNA-free” ~15S subcomplex with a ~30 kDA RNA binding cofactor and, potentially, with other associated cofactors (AFs). The editing domain in mRNAs typically spans multiple blocks (e.g., B1-to-B4), each directed by a gRNA. Transient contacts between RECC and MRB complexes imply that catalysis involves multiple rounds of enzyme association and dissociation with the MRB scaffolds. MRB complexes may also control passage of the edited mRNAs to the mitoribosomes.

We previously showed that the MRB1 variants 3010-MRB and REH2-MRB, with differing protein and gRNA composition, bind the mRNA substrates and products of editing [23]. In that study, we also proposed that these complexes serve as scaffolds for the assembly of gRNA-mRNA hybrids and transient but productive contacts with the RECC enzyme. The current studies showed that: (*i*) these MRB1 variants are tied to distinct editing functions, and (*ii*) specific *cis* and *trans* effects by the regulatory RNA helicase REH2 on substrate loading, complex integrity, and editing can be directly studied in the context of MRB1 complexes. Based on our previous report and current new data, we propose an updated model of MRB1 organization and function (Fig 11).

Both 3010-MRB and REH2 MRB complexes contain all types of mRNA molecules expected to be present during editing, including intermediate transcripts, gRNA and critical subunits such as GAP1/2 proteins, indicating that both complexes are competent editing scaffolds. However, the 3010-MRB complex that we originally purified by immunoprecipitation of native MRB3010 [23] appears particularly active in editing. Interestingly, inducible knockdown of MRB3010 was proposed to inhibit early editing [20]. The hypothesis that the 3010-purified MRB is a major scaffold of the editing machinery is based on our observations that this complex contains increased levels of both initiating gRNAs and mRNAs with increased editing directed by the initiation gRNA. The observed REH2 effects on the RNA profile in 3010-MRB and the integrity of REH2-MRB illustrate how other regulatory proteins may contribute to the editing process. Overall, these data represent the first examples of specific *cis* and *trans* effects by a regulatory helicase on the higher-order RNA editing holoenzyme or “editosome”. REH2, the lone confirmed subunit of MRB1 with a conserved helicase domain, may be a chaperon or remodeling factor that impacts multiple aspects of RNA editing.

We anticipate the characterization of additional MRB1 variants that carry gRNA, GAP proteins, and mRNAs that require editing. Distinct MRB1 variants may control substrate specificity (either loading or stability), and may control different steps during editing or post-editing, including the association of mitoribosomes with the editosome (e.g., as in Fig 5). Specialized or preferential roles of distinct MRB1 variants may depend on the associated “variable” protein subunits, such as REH2, specific protein-RNA interactions, stoichiometric differences of “core” proteins, or combinations of some of these differences. The stoichiometric differences of “core” proteins most likely include MRB3010 (“3010”), which was found to interact with GAP1, but not GAP2, in a yeast two-hybrid screen [37]. Thus, it is possible that 3010 is underrepresented, if not missing, in the protein core of the REH2-MRB. We expect (1) that MRB1 variants that carry gRNA will also contain GAP proteins and (2) that 3010 is necessary for the efficient editing of the associated mRNAs.

Some of the ancillary proteins of the editosome may include known MRB1 components, but not gRNA or GAP proteins. An example is the proposed ~15S REH2 subcomplex including a 30 kDa RNA-binding protein that we described (Figs 7–9). TbRGG2, and the paralogs MRB4160 and MRB8170, that have been detected in most purifications of MRB1, were also found in a separate subcomplex [44]. Interestingly, MRB4160 and MRB8170 interacted with each other in a yeast two-hybrid screen. However, their association with each other *in vivo* is critically dependent on RNA [22,37]. This underscores the importance of stabilizing RNA-protein interactions in the function and regulation of MRB1.

The association of REH2 with an MRB1 variant that contains reduced or no 3010 is puzzling. The stable association of REH2 with GAP proteins and, presumably, other common components of its MRB likely occur via mRNA, gRNA, or both. In addition, the observed transient functional contacts of the REH2 helicase with 3010-MRB may be bridged by mRNA-gRNA hybrids. Both types of REH2 interaction may rely on a coordinated action of the catalytic and accessory domains in this protein. Notably, although depletion of REH2 decreases the loading of the mRNA substrate, it does not dissociate the mRNA already engaged in editing. Nevertheless, additional transient REH2 interactions with 3010-MRB occur during block 1 editing and editing progression through upstream blocks. Putative cofactors of REH2 in the proposed ~15S REH2 subcomplex may influence the interaction and function of conserved domains in REH2 with both the RNA and proteins partners during editing. Interestingly, a putative helicase (Tb927.4.3020) with the same domain organization as REH2 was previously identified among several RNase-sensitive proteins that copurified with REH2 [19,40]. However, a knockdown construct of this protein did not induce an evident editing phenotype, suggesting at least a partial functional redundancy with REH2 (data not shown). A recent study by the Aphasizhev lab confirmed our report that MRB1 complexes contain mRNA editing substrates and products, and also proposed the interesting concept that the subunits of MRB1 complexes, related to 3010-MRB in our studies, may be functionally partitioned in subgroups based on their role in editing or polyadenylation [24]. Several central aspects of RNA editing in kinetoplastids need to be studied including the control of gRNA loading and the transient assembly and activity of RECC complexes in the MRB scaffolds. It seems that editing progression may involve multiple rounds of transient contacts of the RECC enzyme with the MRB1 scaffods, rather than a stable processive interaction. Also, the presence of fully edited mRNA in MRB1 complexes suggests that these complexes mediate the handoff of translatable mRNA into mitoribosomes. Overall, our current observations offer a conceptual framework to undertake systematic studies of the regulation of RNA editing by MRB1 complexes.

## Supporting Information

S1 Fig Editing initiation of mRNA ND7 in native 3010-MRB and REH2-MRB. cDNA sequence of block 1 in mRNA ND7 3’ domain amplified from (A) 3010 and (B) REH2 IPs. PCR primers (arrows) flanking the first block target 5’ unedited and 3’ never-edited sequence. Precursor unedited or edited mRNA (boxed) with editing sites 1-to-8: unedited (gray) or with deletions ‘★’ and insertions ‘**t**‘. Miss-edits (in number or site) are also shown in gray. Edited sequence in block 1 is consistent with the guide domain of gRNA gND7 B1 [gND7(1269–1319)] identified in recent reports in procyclic strains Lister 427 and EATRO 164. A previously annotated encoded T in the ND7 gene (arrow) was missing in all 10 cDNA clones examined here from the Lister 427 strain.
(TIF)

S2 Fig Homology model of motif I mutations. The mutated sites G1365A/K1366Q are shown with the carbons colored white. These mutations in the P loop or motif I (atoms of motif I are shown as sticks) remove one H-bond between beta phosphate of the ADP and REH2. Four H-bonds remain after the mutations.
(TIF)

S1 Table Oligonucleotide primers designed in this study. We designed the indicated primers to perform RT-qPCR of 3’ early editing sites, manual sequencing of block 1 sites, RT-PCR of the first ~200 bp of editing domain (A6 and ND7), RNA interference (RNAi), point mutations and generation of photo-reactive gA6 B1.alt. RT-PCR primers to amplify the entire RPS12 were as in [20].
(TIF)
