# Supplementary figures and images for: Native Variants of the MRB1 Complex Exhibit Specialized Functions in Kinetoplastid RNA Editing
Source: PLoS One. 2015 Apr 30;10(4):e0123441. doi: 10.1371/journal.pone.0123441 (PMC4415780; doi:10.1371/journal.pone.0123441)

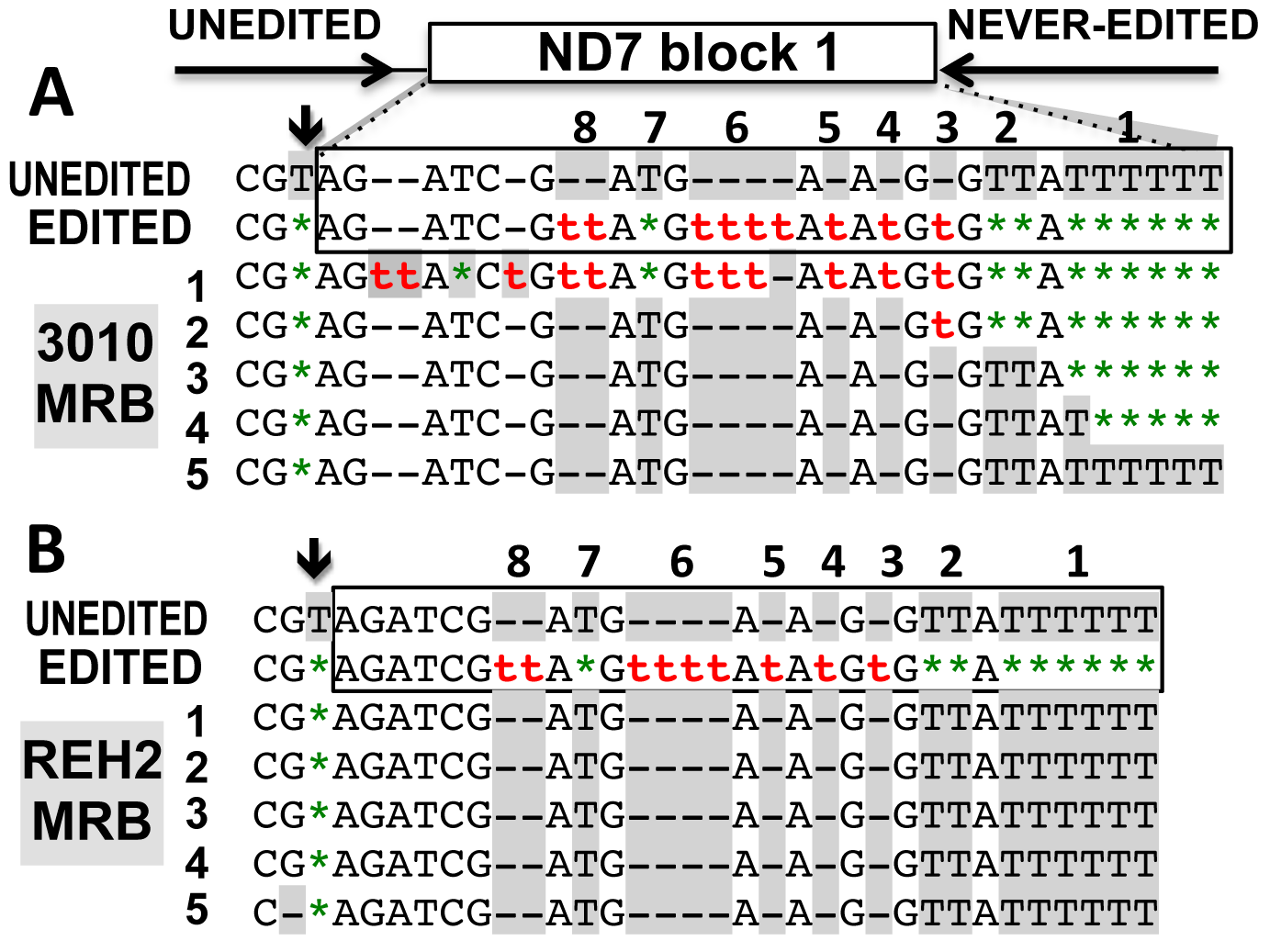

Supplement: S1 Fig — cDNA sequence of block 1 in mRNA ND7 3’ domain amplified from (A) 3010 and (B) REH2 IPs. PCR primers (arrows) flanking the first block target 5’ unedited and 3’ never-edited sequence. Precursor unedited or edited mRNA (boxed) with editing sites 1-to-8: unedited (gray) or with deletions ‘★’ and insertions ‘t‘. Miss-edits (in number or site) are also shown in gray. Edited sequence in block 1 is consistent with the guide domain of gRNA gND7 B1 [gND7(1269–1319)] identified in recent reports in procyclic strains Lister 427 and EATRO 164. A previously annotated encoded T in the ND7 gene (arrow) was missing in all 10 cDNA clones examined here from the Lister 427 strain. (TIF) [file pone.0123441.s001.tif]

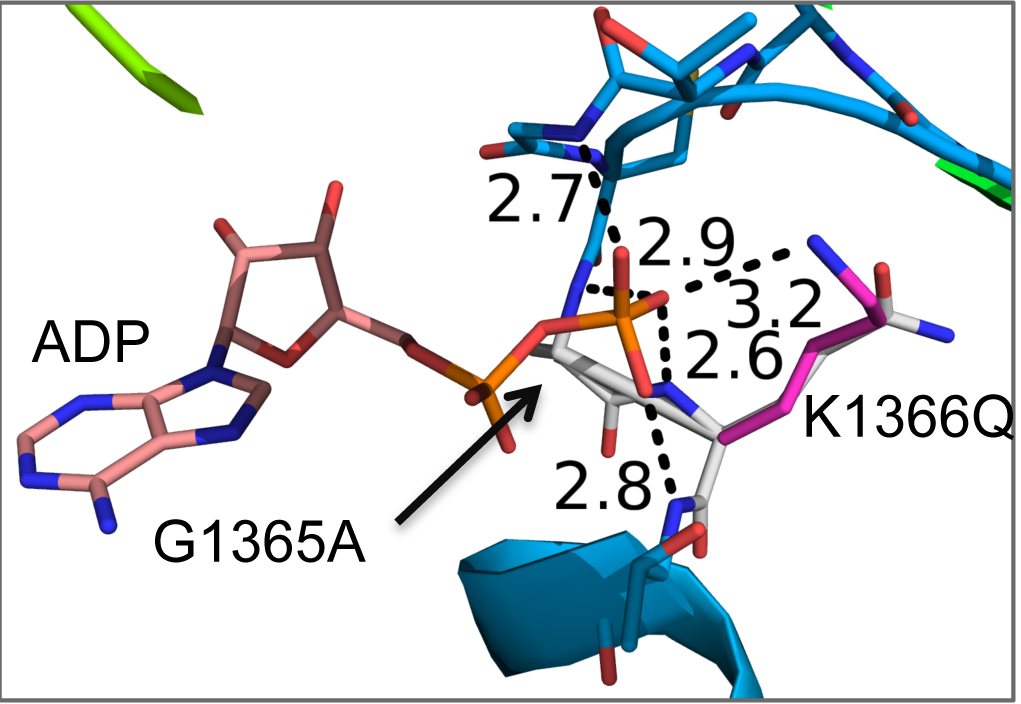

Supplement: S2 Fig — The mutated sites G1365A/K1366Q are shown with the carbons colored white. These mutations in the P loop or motif I (atoms of motif I are shown as sticks) remove one H-bond between beta phosphate of the ADP and REH2. Four H-bonds remain after the mutations. (TIF) [file pone.0123441.s002.tif]

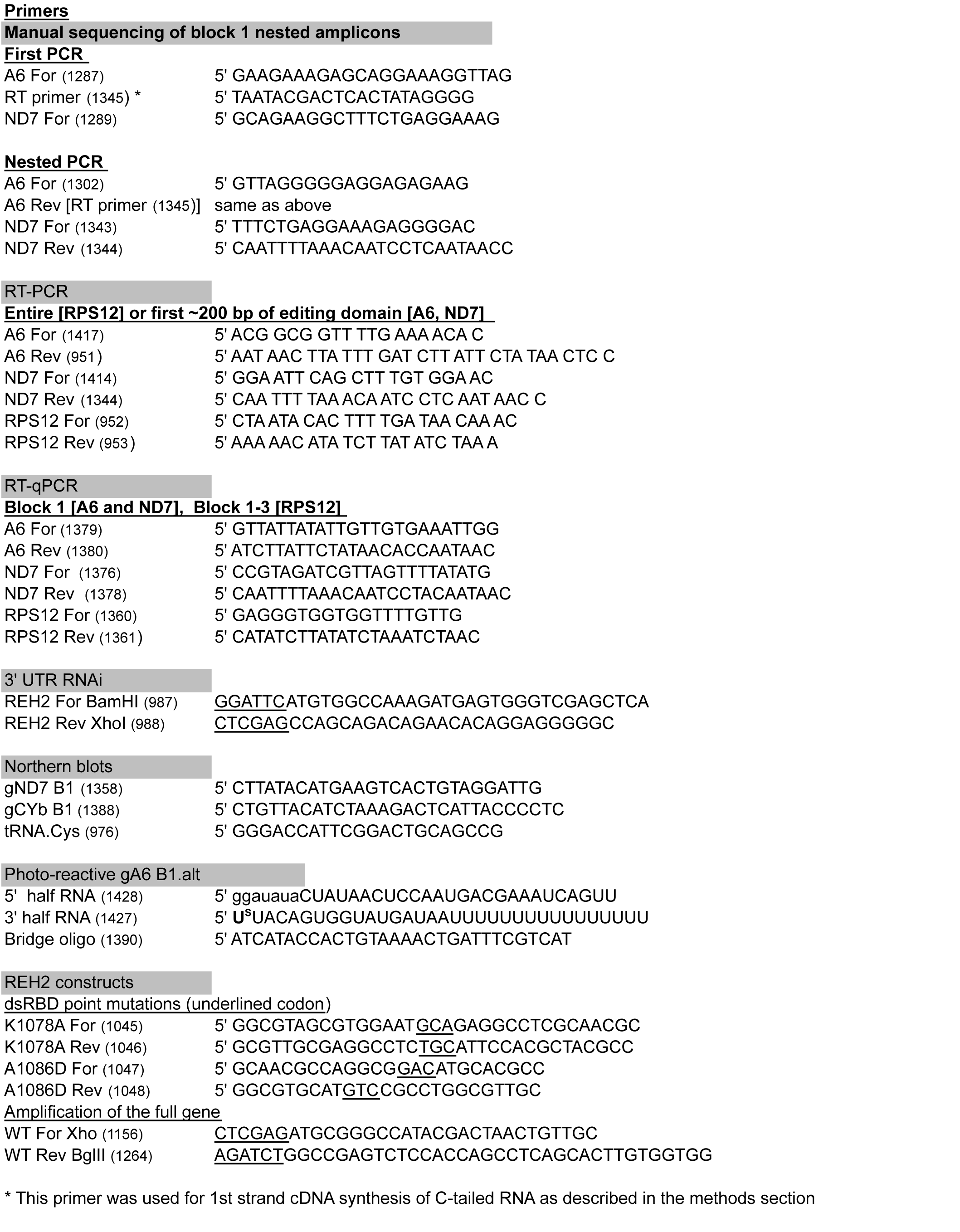

Supplement: S1 Table — We designed the indicated primers to perform RT-qPCR of 3’ early editing sites, manual sequencing of block 1 sites, RT-PCR of the first ~200 bp of editing domain (A6 and ND7), RNA interference (RNAi), point mutations and generation of photo-reactive gA6 B1.alt. RT-PCR primers to amplify the entire RPS12 were as in [20]. (TIF) [file pone.0123441.s003.tif]
